# Reverse Takotsubo cardiomyopathy in the setting of small bowel obstruction: a case report

**DOI:** 10.1097/MS9.0000000000002368

**Published:** 2024-07-17

**Authors:** Daniel Bishev, Hussein Noureldine, Fernando Ortiz

**Affiliations:** a University of Central Florida College of Medicine, Orlando; b HCA Florida North Florida Hospital, Graduate Medical Education Internal Medicine Residency Program; c The Cardiac and Vascular Institute, Gainesville, FL, USA

**Keywords:** heart failure, microvascular dysfunction, stress cardiomyopathy

## Abstract

**Introduction and importance:**

Stress cardiomyopathy refers to a syndrome of acute but reversible left ventricular dysfunction, often triggered by emotional or physical stress. Reverse Takotsubo cardiomyopathy is an uncommon variant that occurs in about 5% of cases. Classically, it has been known to be following a catecholamine surge due to physical or emotional stress. This case highlights the importance for physicians to be aware of the possibility of developing stress cardiomyopathy in patients with acute intra-abdominal processes.

**Case presentation:**

Forty-one-year-old Caucasian female with was admitted with an acute small bowel obstruction. After failing conservative management, it was decided to proceed with surgery. After induction with anesthesia but prior to the surgeons first incision, the patient developed a tachyarrhythmia with hemodynamic compromise requiring the surgery to be aborted. That evening, she developed chest pain with concerns for an acute coronary syndrome. She was taken urgently to the for invasive angiography, which demonstrated reverse Takotsubo.

**Clinical discussion:**

Intra-abdominal processes and intubation have previously been reported be catalyst for this disease process. This patient had multiple stressors including mechanical bowel obstruction and anesthesia after failing conservative management. The diagnosis was confirmed by coronary angiography and left ventriculogram, and followed up with repeat outpatient echocardiography.

**Conclusion:**

A case of small bowel obstruction that developed reverse Takotsubo preceded by sustained ventricular tachycardia after intubation. The patient did well and had complete recovery cardiac function. Risk factors and underlining mechanism for the different variants of stress cardiomyopathy are not well understood, further investigation is warranted.

## Background

Highlights

- The basal variant of Takotsubo cardiomyopathy, also known as reverse or inverted Takotsubo is an uncommon presentation with an estimated prevalence of less than five percent of all cases of stress cardiomyopathy. In this case, it occurred after our patient failed conservative management for small bowel obstruction.
- Acute intra-abdominal pathologies requiring surgery can lead to a catecholamine surges that cause stress cardiomyopathy, and in specific patients, potentially leading to hemodynamic compromise. Physicians should be aware of this when treating patients with these conditions.
- Catecholamine surge is a common but not essential trigger to stress cardiomyopathy, future studies need to be done to investigate the role of vascular spasticity and microvascular dysfunction.

Stress cardiomyopathy refers to a syndrome of acute but reversible left ventricular (LV) dysfunction, often triggered by emotional or physical stress. Clinical presentation of stress cardiomyopathy can mimic acute coronary syndrome. The incidence of stress cardiomyopathy in all troponin-positive patients presenting with suspected acute coronary syndrome (ACS) is about 1–2%. Takotsubo or apical ballooning is the most common variant (75–80%) of stress cardiomyopathies, with the classical appearance of apical hypokinesis or akinesis with basal segments hyperkinesis on echocardiography or left ventriculogram. The basal variant, also known as reverse or inverted Takotsubo is an uncommon presentation with an estimated prevalence of less than five percent of all cases of stress cardiomyopathy. Reverse Takotsubo is defined by the presence of basal hypokinesis with the normal function of the mid-apical myocardium^1^.

The following is a case of reverse Takotsubo in a patient with ventricular arrythmias just prior to bowel surgery. This article was written under the SCARE checklist guidelines^2^.

It is important to diagnose this condition correctly as it can have a significant economic burden. A study published in Clinical Cardiology examined the etiologies, predictors, and economic impact of readmissions within 1 month between years 2013–2014 from the National Readmissions Database. It concluded that although the overall readmission rate was low, the annual national cost impact for index admission and 1‐month readmissions was about $112 million USD^3^.

## Case

A 41-year-old Caucasian female with a past medical history of a cleft palate, abnormal uterine bleeding status post hysterectomy with tubal ligation 1 year prior, who presented to the emergency department with of one day of worsening abdominal pain, nausea, and bilious emesis. Her vitals on presentation were blood pressure of 139/79 mmHg with a heart rate of 88 beats per min, respiratory rate of 18 breaths per min with a peripheral saturation of 100% on room air, and she was afebrile. Laboratory results were only significant for leukocytosis with a white cell count of 15.8 thousand per microliter. Electrocardiogram (ECG) on admission showed sinus bradycardia. Computerized tomography imaging of the abdomen revealed a partial small bowel obstruction (SBO). After conservative management laparotomy was planned on hospital day five.

The patient received standard anesthesia induction with intravenous midazolam (2 mg), propofol (200 mg), fentanyl (50 mcg), and vecuronium (1 mg). Shortly after induction, prior to incision, the patient developed a narrow complex tachycardia that became wide with a heart of 187 bpm. She also experienced a hypertensive response with blood pressure rising to 204/120 mmHg. She was given one 100 mg of intravenous lidocaine with termination of the wide complex tachycardia. High-sensitivity (HS) troponins checked immediately after the event were minimally elevated at 35 (pg/ml, normal range < 34). Intraoperative transesophageal echocardiogram revealed a dilated left ventricular cavity with reduced systolic and diffuse hypokinesis. Moderate tricuspid and mitral regurgitation were also noted. At this time, the surgery was aborted, and the patient was returned back to the medical wards for further evaluation and cardiac monitoring.

Later that day she developed sudden onset substernal chest pain, which was relieved by sublingual nitroglycerin. Serial HS troponins were obtained and peaked at 4437 pg/ml. Repeat ECG showed normal sinus rhythm with a prolonged QTc at 493 ms. Non-ST-segment elevation myocardial infarction (NSTEMI), Spontaneous coronary artery dissection, stress cardiomyopathy and myocardial infarction with non-obstructive coronary arteries (MINOCA) were on the differential and a left heart catheterization was planned to determine the definitive diagnosis.

**Figure 1:**
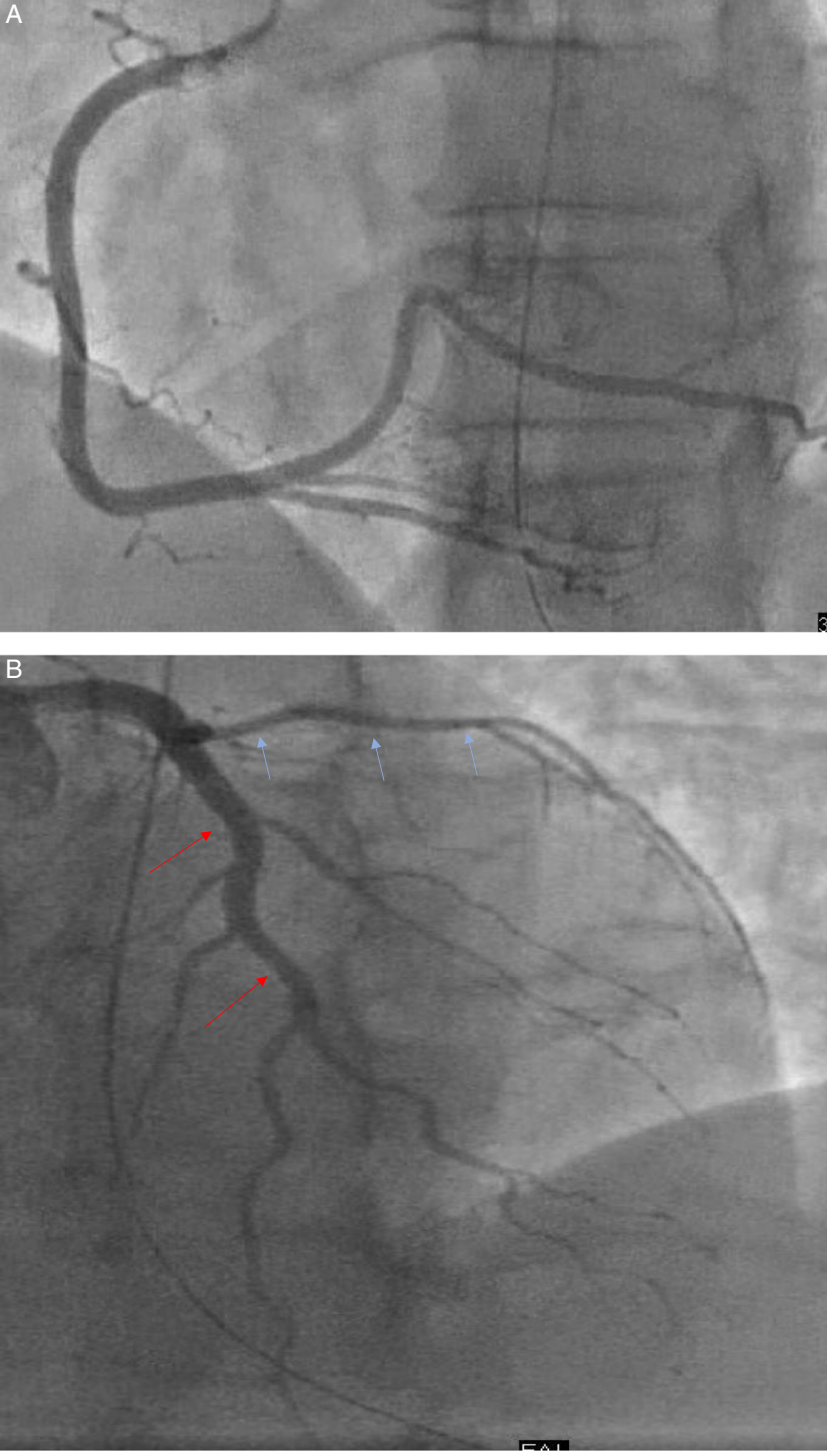
(A) Right coronary branches on angiography showing patent arteries. (B) Left coronary branches on angiography showing patent.

On hospital day 6 she underwent coronary angiography, which revealed widely patent coronary arteries with minimal disease (Fig. 1). Left ventriculogram showed a hyperkinetic apex with hypokinesis of basal to mid segments confirming the diagnosis of basal variant stress cardiomyopathy or reverse Takotsubo. The estimated ejection fraction was 45%. Guideline directed medical therapy was recommended but unfortunately the patient could only tolerate low-dose beta-blockers due to symptomatic hypotension. At her follow-up appointment, a repeat echocardiogram showed complete recovery of left ventricular function; with an ejection fraction of 60-65%, no wall motion abnormalities, and normal global longitudinal strain.

## Discussion

The hypothesis for the cause of reverse stress cardiomyopathy in this case is based on multiple stressors that cumulatively led to a state of catecholamine excess. The exact mechanism of stress cardiomyopathy is still unknown. The two currently predominant theories include (1) a central autonomic nervous system-mediated activation of specific neuropeptides, and (2) an increase in circulating catecholamines. Both theorized mechanisms are an end result of excessive emotional or physical stressors^4^. In some individuals during times of heightened emotional or physical stress, there is excess release of norepinephrine (NE) and stress-related neuropeptides as neuropeptide Y in the synaptic junction of the sympathetic nerves and myocardium. The rapid accumulation of these neuropeptides the myocardial level is suspected to have a toxic effect, which may lead to epicardial and microvascular dysfunction^5,6^. The transient state of epicardial and microvascular dysfunction results in reduced contractility or “Myocardial Stunning”^6^. Alternatively, it is speculated that stunned myocardium is a result of circulating catecholamines such as epinephrine and NE as opposed to localized release of neuropeptides at neurons overlying the myocardium^6^. However, the release of circulatory catecholamines in stress cardiomyopathy could be a compensatory mechanism to decreased cardiac output and hypotension rather than the primary trigger of myocardial dysfunction^7^.

It is important to note, NE and NPY are a part the normal physiological response to stress and do not cause stunning or decreased contractility. It is possible that individuals vulnerable to stress cardiomyopathy also have underlining microvascular dysfunction which in conjunction with excess neurohormonal exposure results in a demand-supply perfusion mismatch triggering transient ischemic stunning^8^. This theory is supported by the observation that stress cardiomyopathy and microvascular dysfunction share multiple common risk factors (post-menopausal state, asthma, chronic obstructive disease, diabetes, substance use disorder)^9^. Further studies are needed to elucidate the mechanism of stress cardiomyopathy and to identify those at risk.

Reversible LV apical hypokinesis has been induced in rats’ models with the administration of intravenous catecholamines and exposure to emotional stressors. These findings suggest an association between emotional stressors or catecholamine surges, and wall motion abnormalities^9^. Bolli demonstrated that cellular free radical accumulation resulted in myocyte dysfunction myocardial stunning, a sublethal form of oxyradical-mediated “reperfusion injury. Free radical accumulation is a result of excess cyclic adenosine monophosphate production caused by over-activation of beta-2 receptors from catecholamines^10^. The persistence of such free radicals would trigger the expression of genes that eventually induce apoptosis^11^. However, apoptosis, which implies permanent damage to the myocardium is not a common finding in stress cardiomyopathy and thus it is believed that free radicals are cleared before apoptosis is induced. Clearance of free radicals is achieved by intrinsic cellular antioxidant factors, which eventually reverses the myocardial dysfunction^11^.

Additionally, a study investigating hemodynamic changes in response to intubation and laryngoscopy found a significant surge in catecholamines in patients. Norepinephrine surges during laryngoscopy when induction of anesthesia was achieved with thiopentone were observed with no notable change in the levels of other catecholamines^12^. The study included 36 patients undergoing minor orthopedic or gynecological surgeries, which were intubated under standardized laryngoscopy procedure with no adverse events reported^12^. The findings of this study suggest that catecholamine surges can be observed with sympathetic stimulation caused by laryngoscopy^13^. This could explain the sudden deterioration of our patient during intubation. However, in this study, pancuronium was the paralytic of choice for all participants^12^. A study by Pauca and Skovsted demonstrated that pancuronium increases blood pressure and pulse pressure in 31 patients undergoing surgery without a history of cardiopulmonary disease, but the study did not evaluate the effect of the paralytic on catecholamines^14^. Thus, the association between intubation and catecholamine is still unclear in the presence of cofounders such pancuronium.

This case demonstrates the resultant stress cardiomyopathy after a small bowel obstruction that required surgery. The patient experienced multiple stressing events including an acute bowel obstruction, induction with anesthetic and paralytic agents, and all of this followed by intubation caused the patient to develop reverse Takotsubo cardiomyopathy. The cardiomyopathy is explained by catecholamine surge; however, she may have also been at increased risk. Estrogen has been shown to play a role in vascular relaxation^15^. In this patient who had a history of abnormal uterine bleeding, the likelihood of developing a stress cardiomyopathy may have been higher. For this reason, physicians should be aware of this when treating patients with acute intra-abdominal processes.

## Conclusion

Stress cardiomyopathy is a reversible cardiomyopathy with multiple variants, which can be triggered by physical or emotional stress. Intra-abdominal processes and intubation have previously been reported be catalyst for stress cardiomyopathy. We presented a case of a 41-year-old female with SBO that developed the rare basal variant, which manifested with sustained ventricular tachycardia after intubation. Ultimately our patient did well and had complete recovery cardiac function. Risk factors and underlining mechanism for the different variants of stress cardiomyopathy are not well understood, further investigation is warranted.

## Ethical approval

Not applicable.

## Consent

Available upon request.

## Source of funding

This research did not receive any specific grant from funding agencies in the public, commercial, or not-for-profit sectors.

## Author contribution

D.B. (primary investigator), H.N. (co-author), F.O. (co-author).

## Conflicts of interest disclosure

The authors declare no conflicts of interest.

## Research registration unique identifying number (UIN)

Not applicable.

## Guarantor

Not applicable.

## Data availability statement

Not applicable.

## Provenance and peer review

not commissioned, externally peer-reviewed

## Disclaimer

This research was supported (in whole or in part) by HCA Healthcare and/or an HCA Healthcare affiliated entity. The views expressed in this publication represent those of the author(s) and do not necessarily represent the official views of HCA Healthcare or any of its affiliated entities.
